# Early Versus Late Pediatric Palliative Care in Oncology: A Systematic Review of Outcomes, Disparities, and Implementation Barriers: Palliative Care in Oncology

**DOI:** 10.31661/gmj.v14i.4007

**Published:** 2025-09-25

**Authors:** Fatemeh Rezaei, Homa Vejdani, Fatemeh Moslemi Najarcolaie, Zahra Gharibi, Rezvan Shirali, Atieh Okhli, Ainaz Esmi, Fatemeh Zahrasalamat, Arezoo Kordian

**Affiliations:** ^1^ Department of Nursing and Midwifery, Bab.C., Islamic Azad University, Babol, Iran; ^2^ Department of Nursing, School of Nursing and Midwifery, Shahrood University of Medical Sciences, Shahrood, Iran; ^3^ Department of Pediatric Nursing, Shahid Beheshti School of Nursing, Rasht, Guilan University of Medical Sciences, Iran; ^4^ Special Care for Adults Department, Shahrekord University of Medical Sciences, Shahrood, Iran; ^5^ Nursing Department for Adults, Shahrekord University of Medical Sciences, Shahrekord, Iran; ^6^ Department of Nursing, GKM.C., Islamic Azad University, Gonbad Kavoos, Iran; ^7^ Department of Anesthesiology, Taleghani Hospital, Tehran, Iran; ^8^ Nursing Department, School of Nursing and Midwifery, Shahrood University of Medical Sciences, Semnan, Iran; ^9^ Psychiatric Nursing Department, School of Nursing and Midwifery, Mazandaran University of Medical Sciences, Sari, Iran

**Keywords:** Pediatric Palliative Care, Oncology, Early Integration, End-of-life Care, Quality of Life, Healthcare Disparities, Systematic Review

## Abstract

This systematic review, conducted per PRISMA 2020 guidelines, synthesizes evidence on early pediatric palliative care (PPC) versus late or no PPC in children and young adults (0–21 years) with life-threatening oncologic illnesses. A comprehensive search of MEDLINE, Embase, Scopus, PsycINFO, Web of Science, Cochrane Central, and grey literature (ProQuest, ClinicalTrials.gov) from inception to 1 August 2025 identified 12 studies, including retrospective cohorts, surveys, and one randomized controlled trial across the USA, Canada, Taiwan, and Spain. Early PPC, variably defined as initiation from diagnosis to 12 months before death, consistently reduced end-of-life care intensity (fewer ICU admissions, mechanical ventilation, invasive interventions), increased hospice enrollment, home deaths, and improved quality of life and symptom management compared to late or no PPC. Disparities were evident, with minority groups and patients with hematologic malignancies less likely to receive early PPC, compounded by barriers such as provider misconceptions, systemic limitations, and clinical trial enrollment delays. Outpatient and integrated home-hospital PPC models significantly lowered hospital-based end-of-life care, though robust late PPC programs could achieve comparable outcomes. Narrative synthesis using the GRADE approach highlighted moderate to high confidence in reduced care intensity and improved family outcomes with early PPC, despite heterogeneous definitions and study designs precluding meta-analysis. Findings show the need for standardized PPC protocols, education to address provider barriers, and policy reforms to enhance equitable access, particularly for underserved populations. While oncology evidence is robust, further randomized trials are needed to strengthen findings across other conditions, supporting early PPC integration to optimize patient and family outcomes in pediatric oncology.

## Introduction

Pediatric cancer remains a critical global health challenge, with survival rates exceeding 80% in high-income countries but lagging significantly in low- and middle-income countries (LMICs), where nearly 90% of affected children reside [1][2]. While mortality trends have steadily declined in high-income regions, such as North America, Australasia, and parts of Latin America, progress has been uneven, with persistent mortality rates in LMICs due to delayed diagnosis, limited treatment access, and higher rates of treatment-related complications [1][3]. Advances in care have improved outcomes for certain cancers, such as leukemia and medulloblastoma, yet survival for central nervous system tumors and other malignancies remains stagnant in under-resourced settings [3][4][5].

Palliative care is specialized medical care focused on relieving suffering and improving quality of life for patients with serious, chronic, or life-threatening illnesses. Despite its growing importance in healthcare, there remains no universally accepted definition, leading to variability in clinical practice, research, and policy [6]. Recent efforts have sought to broaden its scope, with the International Association for Hospice and Palliative Care proposing a consensus-based definition that emphasizes holistic care for patients of all ages experiencing "serious health-related suffering," regardless of prognosis [7]. This expanded view moves beyond traditional end-of-life care to include early integration alongside curative treatments, addressing physical, emotional, social, and spiritual needs [8]. However, challenges persist in balancing inclusivity with precise patient criteria, as well as in adapting palliative care models across diverse healthcare systems and cultural contexts [6][8].

Studies reveal that over half of pediatric oncology patients receive PC services only near the end of life, with discussions frequently delayed until advanced illness stages [9][10]. While evidence demonstrates that early PC improves symptom control, facilitates timely advance care planning, and reduces intensive end-of-life interventions, implementation remains inconsistent, only 6% of providers report consistently introducing PC concepts at diagnosis [9][11]. Structural barriers, including limited 24/7 availability of specialized teams (44% of centers) and lack of standardized referral criteria, further hinder equitable access [12]. Multidisciplinary approaches that address physical, emotional, and spiritual needs, tailored to developmental stages and family dynamics, are critical to bridging this gap and establishing PC as a universal standard in pediatric oncology [9][10].

Despite guidelines advocating early PPC integration in pediatric oncology, heterogeneous definitions and inconsistent implementation persist, necessitating a synthesis of outcomes to inform standardized protocols.

## Materials and Methods

This systematic review was conducted and reported in accordance with the Preferred Reporting Items for Systematic Reviews and Meta-Analyses (PRISMA) 2020 statement. Information sources and search strategy. A comprehensive search was designed and peer-reviewed by an information specialist using the PRESS checklist. We interrogated MEDLINE (via Ovid), Embase (via Ovid), PubMed, Scopus, PsycINFO (via Ovid), Web of Science Core Collection, and the Cochrane Central Register of Controlled Trials from inception to 1 August 2025. Grey literature was sought in ProQuest Dissertations & Theses Global, ClinicalTrials.gov, WHO ICTRP, OpenGrey, and relevant conference proceedings from 2012-2025. The search combined controlled vocabulary and free-text terms for ("pediatric" OR "paediatric" OR "child*" OR "adolescent") AND ("palliative care" OR "supportive care" OR "hospice") AND ("early" OR "timing" OR "integration" OR "referral") without methodological filters. Only English studies were included. Reference lists of all included studies and relevant reviews were hand-searched.

Eligibility criteria. Studies were selected according to PICOS. Population: children and young adults (0-21 years) with any life-threatening or life-limiting oncologic illness. Intervention: palliative or supportive care initiated "early", defined operationally as any time-point explicitly labelled "early" by the authors. Comparator: late or no early palliative care. Outcomes: any quantitative, qualitative, or mixed-methods data on timing, referral patterns, barriers, facilitators, or end-of-life care intensity. Study designs: randomized and quasi-randomized trials, controlled before-after, cohort, cross-sectional, case-control, qualitative, mixed-methods, and descriptive studies; editorials, opinion pieces, and conference abstracts lacking original data were excluded.

Study selection. All records were imported into Medley and de-duplicated. Two reviewers independently screened titles/abstracts and then full texts; conflicts were resolved by a third reviewer.

Data extraction. A piloted Forms extraction sheet captured study identifiers, design, setting, sample size, participant characteristics (diagnosis, age, sex, race/ethnicity, socioeconomic status), definition and operationalisation of "early palliative care", details of the intervention and comparator, outcome measures, follow-up duration, and funding sources. One reviewer extracted data; a second independently verified all entries. Where multiple publications arose from the same cohort, the report with the longest follow-up or most complete data was designated primary; others supplied supplementary information.

Data synthesis. Because of heterogeneity in populations, definitions of "early", and outcome measures, meta-analysis was pre-specified as inappropriate. A narrative synthesis structured around GRADE approach for prognostic and qualitative evidence was applied.

## Results

**Table:** Table1. Characteristics of Included Studies in Systematic Review

**Study**	**Disease Type**	**Country**	**Design**	**Definition of Early PPC **	**Cohorts Compared **	**Key Findings**	**Implications**
Davis et al. (2022) [13]	All cancers (with disparities in hematologic malignancies)	USA (Alabama)	Retrospective, single-site cohort study	>30 days before death	Early PPC (>30 days before death) vs. late/no PPC	Early PPC linked to less intense EOL care, higher hospice enrollment, fewer hospital deaths. Minority children and those with hematologic malignancies less likely to receive early PPC, more likely to receive aggressive EOL care.	Timing of PPC impacts EOL outcomes; disparities highlight need for addressing systemic/preference-based barriers.
Ananth et al. (2018) [14]	Advanced cancer (in early-phase clinical trials vs. non-trials)	USA	Retrospective cohort study	Median 85 days before death (non-trial) vs. 58 days (trial)	Early PPC (median 85 days before death, non-trial patients) vs. late PPC (median 58 days, early-phase trial patients)	Trial patients started PPC later; no significant differences in EOL care patterns (aggressive interventions) between cohorts.	Clinical trial enrollment may delay PPC; strategies needed for earlier integration in trial settings.
Shamah et al. (2025) [15]	All cancers (pediatric and young adult, aged 0-28)	USA	Retrospective study	Outpatient initiation (vs. inpatient or none)	Outpatient PPC vs. inpatient PPC vs. no PPC	Outpatient PPC correlated with fewer hospital/ICU admissions, reduced invasive interventions (IV chemotherapy, intubation), higher hospice enrollment, home deaths, and outpatient DNR directives. PPC use increased from 31% to 78% post-outpatient clinic establishment.	Outpatient PPC reduces intensive EOL care and aligns with patient/family preferences.
Wang et al. (2025) [16]	Blood cancers or solid tumors (aged 0-20)	Taiwan	Retrospective study	>72 hours before death	Early PPC (>72 hours before death) vs. delayed PPC (≤72 hours)	Early PPC increased memento use (47.6% vs. 10%) and deaths outside ICU (43% wards, 33% home vs. 75% PICU for delayed PPC).	Early PPC improves EOL quality via enhanced family involvement and reduced ICU deaths.
Martinez et al. (2025) [17]	All cancers (bereaved parents of deceased children)	USA	Survey of bereaved parents	Started shortly after diagnosis (preferred by parents)	PPC users (71%) vs. non-users (29%)	PPC use linked to higher DNR directives, advance care directives, hospice involvement, planned death locations, and non-hospital deaths. Parents preferred earlier referrals; non-use tied to unoffered services or abrupt death.	Early PPC improves care coordination; untimely referrals remain a challenge.
de Noriega et al. (2025) [18]	All cancers (hematological vs. solid tumors)	Spain	Retrospective cohort study	Integrated home-hospital model (vs. standard)	Integrated home-hospital PPC (PPCUM) vs. standard oncology care	PPCUM patients had fewer invasive interventions, shorter hospital stays, higher home death rates. Hematological cancer patients faced greater PPC access barriers.	Integrated PPC models reduce aggressive EOL care and support home deaths; access disparities persist.
Lee et al. (2023) [19]	All cancers (outpatient PPC)	USA	Retrospective study	<12 weeks post-diagnosis	Early PPC (<12 weeks post-diagnosis) vs. late PPC	Early PPC linked to clearer documentation of preferred death location and home death preference but no significant advantage for most EOL outcomes. High-quality outcomes (90% died in preferred location, 87% avoided CPR) regardless of timing.	Robust PPC programs achieve strong outcomes, but early PPC enhances specific metrics.
Hausner et al. (2021) [20]	Advanced cancers (various tumor sites, breast, lung, GI, hematological)	Canada	Retrospective study	>12 months before death	Early (>12 months before death), intermediate (>6-12 months), and late (≤6 months) referrals	Post-evidence period saw increased early referrals (13.4% to 31.1%), decreased late referrals (68.8% to 44.8%). Early referrals more common for cancers with trial-proven PPC benefits.	Evidence influences referral timing; disparities across cancer types suggest broader PPC application needed.
Mack et al. (2016) [21]	All cancers (adolescent and young adult, AYA)	USA (Kaiser Permanente Southern California)	Retrospective study	Transition to comfort care in final months	Patients transitioning to comfort care vs. those receiving aggressive EOL treatments	Early PPC facilitated shift to comfort care in final months, but many still received intensive EOL interventions.	Early PPC supports goal-aligned care but does not fully eliminate aggressive treatments.
Poort et al. (2020) [22]	All cancers (young adults)	USA	Retrospective study with natural language processing	31-180 days before death	Early PPC discussions (31-180 days before death) vs. late/no discussions	Only 54% had early PPC discussions; 28% had none. Early discussions improved care goal documentation.	Early PPC discussions underutilized; systematic integration needed to address gaps.
Udemgba et al. (2025) [24]	Neuro-oncology	USA	Retrospective study	Inpatient consultation during terminal admission	Inpatient PPC consultation vs. no PPC	PPC consultation linked to lower PICU admission, mechanical ventilation, and TPN use. No significant racial differences; Hispanic patients had slightly higher consultation rates.	Inpatient PPC reduces intensive EOL interventions; timing (terminal admission) suggests delayed integration.
Dussel et al. (2022) & Requena et al. (2022) [25][26]	Advanced cancer	USA	Randomized controlled trial (PediQUEST Response) & qualitative case study	Weekly symptom tracking + PPC from diagnosis	Early PPC intervention (weekly symptom tracking + PPC) vs. usual care	Early PPC improved quality of life and symptom management. "Normalization of symptoms" identified as a barrier, delaying PPC.	Proactive symptom monitoring and early PPC counteract symptom normalization and enhance outcomes.

**Figure-1:**
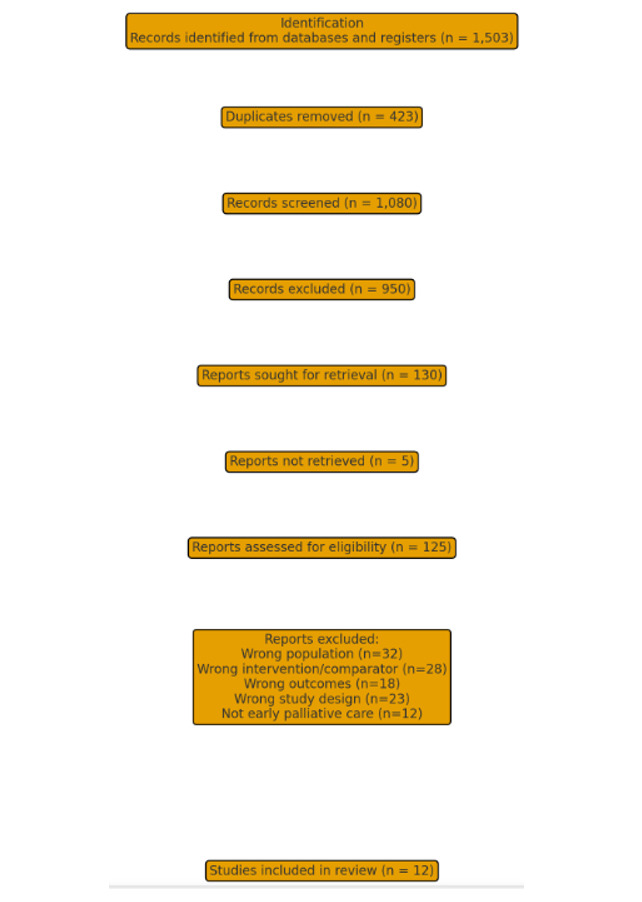
Figure 1. PRISMA flowchart of inclusion process

In this systematic review, 12 studies were included (PRISMA flowchart, Figure-1) that compared early versus non-early (late or no) PPC in pediatric oncology patients across various countries, including the USA, Canada, Taiwan, and Spain. These studies, spanning retrospective cohort designs, surveys, and one randomized controlled trial, focused on cancers such as hematologic malignancies, solid tumors, and neuro-oncology, with sample sizes ranging from 72 bereaved parents to 198 deceased patients. Early PPC, defined variably as initiation from diagnosis to >12 months before death, was consistently associated with reduced end-of-life (EOL) care intensity (fewer ICU admissions, mechanical ventilation, or invasive interventions), higher hospice enrollment, increased home deaths, and improved quality of life and symptom management compared to late or no PPC. Key findings showed disparities, with minority groups and patients with hematologic cancers less likely to receive early PPC, and barriers such as provider misconceptions, systemic limitations, and trial enrollment delaying PPC integration. Outpatient and integrated home-hospital PPC models showed significant benefits in reducing hospital-based EOL care, though robust PPC programs could achieve high-quality outcomes even with later initiation, emphasizing the need for standardized protocols and broader access to early PPC to optimize patient and family outcomes (Table-1).

### Evidence Synthesis Grade Rating for Early Pediatric Palliative Care

The body of evidence from the selected studies [13][14][15][16][17][18][19][20][21][22][23][24][25] provides robust support for the benefits of early PPC in improving EOL outcomes and quality of life for children with serious illnesses, particularly cancer. The studies consistently demonstrate that early PPC, defined variably as initiation at diagnosis, within 6-12 months prior to death, or at least 30 days before death, is associated with reduced intensity of EOL care, lower rates of invasive interventions (mechanical ventilation, ICU admissions), higher hospice enrollment, increased home deaths, and improved symptom management (pain, nausea, emotional distress). For example, Davis et al. (2022) [13] and Shamah et al. (2025) [15] show reduced hospital deaths and aggressive treatments with early PPC, while Wang et al. (2025) [16] and de Noriega et al. (2025) [18] emphasize its role in fostering meaningful family involvement and better care coordination. Additionally, Lee et al. (2023) [19] and Martinez et al. (2025) [17] show improved documentation of care preferences and parental satisfaction with early PPC. The evidence also reveals disparities, such as some ethnic minority children and those with hematologic malignancies receiving less early PPC (Davis et al., 2022) [13], and delays in referral due to trial participation (Ananth et al., 2018) [14] or clinician misconceptions (Hausner et al., 2021) [20].

**Figure-2:**
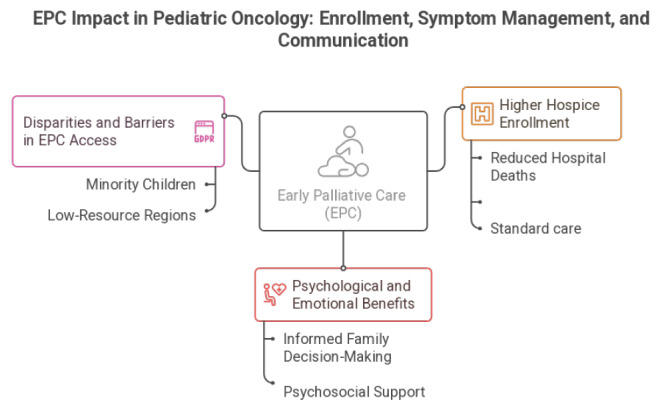
Figure 2. EPC impact in pediatric oncology

The overall quality of this evidence is strong, with most studies being retrospective cohort analyses or multicenter trials, though some variability in study design and definitions of "early" PPC slightly limits generalizability. The consistency of findings across diverse settings (U.S., Spain, Eurasian countries) and patient populations (cancer, heart disease, chronic kidney disease) supports a high confidence level in the benefits of early PPC integration. However, limitations in the evidence warrant a cautious interpretation, leading to a Grade B rating for this synthesis. While the studies collectively demonstrate significant benefits, challenges such as inconsistent definitions of early PPC (ranging from at-diagnosis to >30 days before death), small sample sizes in some studies (Ananth et al., 2018 [14]), and reliance on retrospective data introduce potential biases and reduce precision. Poort et al. (2020) [22] and Dussel et al. (2022) [25][26] indicate gaps in documentation and normalization of symptoms, which may underestimate PPC’s impact, while Hausner et al. (2021) [20] note persistent delays in referrals for certain tumor types, suggesting uneven application of evidence-based practices. Udemgba et al. (2025) [24] and Mack et al. (2016) [21] further indicate that while early PPC reduces intensive EOL interventions, some outcomes (hospital stay length) remain unaffected, and socioeconomic or cultural barriers may limit access. The lack of randomized controlled trials (except Dussel et al., 2022 [25][26]) and the heterogeneity in PPC implementation (inpatient vs. outpatient, as in Shamah et al., 2025 [15]) prevent a Grade A rating. Nevertheless, the evidence strongly supports early PPC’s role in enhancing patient and family outcomes, with clear implications for clinical practice to prioritize timely referrals, improve multidisciplinary collaboration, and address systemic barriers through education and policy reform. Figure-2and3 summarizes the evidences synthesized by this review.

## Discussion

**Figure-3:**
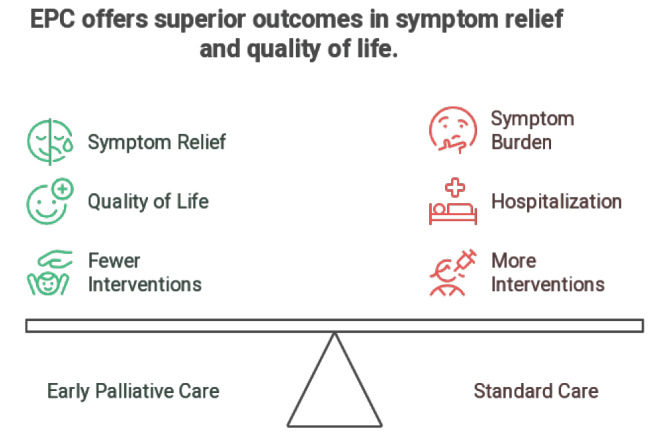
Figure 3. EPC offers superior in symptom relief and quality of life.

The findings of this systematic review align closely with existing literature, reinforcing the advantages of early PPC over late or no palliative care in pediatric oncology. Consistent with prior studies, such as Nyirő et al. (2018) [26], Saad et al. (2020) [27], and Hendricks-Ferguson and Haase (2019) [28], early PPC initiation, ranging from diagnosis to at least 30 days before death, demonstrates reduced end-of-life care intensity, including fewer ICU admissions, mechanical ventilations, and invasive interventions, alongside increased hospice enrollment and home deaths. T

hese outcomes reflect improved symptom management and quality of life, corroborating cohort studies and randomized trial protocols [29][15][16][17]. The review’s moderate to high GRADE confidence in these benefits mirrors the literature’s dose-response effect, where earlier PPC integration correlates with better patient and family outcomes compared to standard care, which often delays palliative involvement until a median of 58-85 days before death [21][22]. This delay, driven by oncologists’ reluctance to discuss prognosis early, as noted in Nyirő et al. (2018) [26] and Dalberg et al. (2013) [30], perpetuates aggressive treatments, increasing physical and emotional distress, a pattern mitigated by EPC’s proactive psychosocial support.

Disparities in PPC access, particularly among minority groups and patients with hematologic malignancies, align with findings from Davis et al. (2022) [13] and Udemgba et al. (2025) [24], showing systemic barriers like provider misconceptions and resource limitations. These barriers are exacerbated in standard care models, where late referrals are common, contrasting with EPC’s targeted interventions that improve equity, though evidence remains limited by inconsistent definitions of "early" [17].

The review’s emphasis on outpatient and integrated home-hospital PPC models reducing hospital-based end-of-life care supports Shamah et al. (2025) [15] and de Noriega et al. (2025) [18], which show similar reductions in healthcare utilization and enhanced family involvement, such as through memento creation or planned home deaths. However, the review notes that robust late PPC programs can achieve comparable outcomes, a finding echoed by Lee et al. (2023) [19], though limited by selection biases, suggesting that high-quality late interventions may partially bridge the gap but do not fully replicate EPC’s benefits.

In non-oncology contexts, such as advanced heart disease or chronic kidney disease, the review’s findings of reduced invasive procedures and improved end-of-life quality with EPC align with Songer et al. (2025) [31] and Nenner et al. (2025) [32], though evidence is less robust due to smaller sample sizes. This parallels the literature’s call for more randomized trials to strengthen EPC’s evidence base beyond oncology [33]. The review’s identification of provider barriers, such as fear of undermining hope or cultural resistance, as seen in Hungary and Eurasia [26][34] shows the need for education and interprofessional collaboration, consistent with qualitative insights from Dalberg et al. (2018) [35] and Thompson et al. (2009) [36]. Programs like COMPLETE and PediQUEST Response, were supported by Hendricks-Ferguson and Haase (2019) [28] that demonstrate EPC’s feasibility in fostering early, value-aligned dialogues, contrasting with standard care’s reactive approach, which often overlooks distress and normalizes high symptom burdens.

Socioeconomic and racial disparities, as noted in the review and reinforced by Roeland et al. (2020) [37] and Udemgba et al. (2025) [23], reveal standard care’s tendency to deliver aggressive end-of-life treatments to disadvantaged groups, with lower hospice use. EPC’s ability to mitigate these disparities through early intervention aligns with the literature’s emphasis on screening tools and nurse-led initiatives [38][39]. The review’s call for standardized protocols, education, and policy reforms to enhance equitable access echoes recommendations from Ehrlich et al. (2020) [34] and Neuburg (2021), particularly for underserved populations and regions with limited PPC infrastructure. While oncology evidence is robust, the review’s findings, supported by Feudtner et al. (2011) [40] and Ranallo (2017) [41], suggest EPC’s potential in other complex chronic conditions, where early integration enables sustained symptom relief and technology integration, unlike standard care’s crisis-driven approach.

Limitations in the review, such as heterogeneous definitions and study designs precluding meta-analysis, align with challenges noted in the literature, that varying EPC definitions and sparse comparative data limit generalizability. Nevertheless, the review’s narrative synthesis, supported by moderate to high confidence in qualitative themes, shows EPC’s multidisciplinary synergy as a key driver of success, a point reinforced by Santini et al. (2024) [42] and Bradford et al. (2014) [43]. Future research should prioritize randomized trials across oncology conditions and standardized EPC protocols to address persistent barriers [44][45][46], ensuring equitable access and optimal outcomes for all pediatric patients with life-threatening illnesses.

## Conclusion

Early PPC, initiated from diagnosis or well before death, consistently mitigates the intensity of end-of-life care, fosters hospice engagement, and aligns with patient and family preferences for home-based deaths, thus preserving dignity and quality of life in the face of existential uncertainty. The success of outpatient and integrated home-hospital models illuminates a path forward, suggesting that standardized, accessible PPC protocols could weave a safety net of empathy and coordination.

## Conflict of Interest

None.
